# Optimization of protein samples for NMR using thermal shift assays

**DOI:** 10.1007/s10858-016-0027-z

**Published:** 2016-03-17

**Authors:** Sandra Kozak, Lukas Lercher, Megha N. Karanth, Rob Meijers, Teresa Carlomagno, Stephane Boivin

**Affiliations:** SPC Facility, European Molecular Biology Laboratory (EMBL), Hamburg Outstation, Notkestrasse 85, 22607 Hamburg, Germany; SCB Unit, European Molecular Biology Laboratory (EMBL), Meyerhofstrasse 1, 69117 Heidelberg, Germany; BMWZ and Institute of Organic Chemistry, Leibniz University Hannover, Schneiderberg 38, 30167 Hannover, Germany; Research Group of NMR-based Structural Chemistry, Helmholtz Centre for Infection Research, Inhoffenstrasse 7, 38124 Brunswick, Germany

**Keywords:** Differential scanning fluorimetry, Nuclear magnetic resonance, Protein thermal stability, Sample optimization, Thermal shift assay, ThermoFluor

## Abstract

Maintaining a stable fold for recombinant proteins is challenging, especially when working with highly purified and concentrated samples at temperatures >20 °C. Therefore, it is worthwhile to screen for different buffer components that can stabilize protein samples. Thermal shift assays or ThermoFluor^®^ provide a high-throughput screening method to assess the thermal stability of a sample under several conditions simultaneously. Here, we describe a thermal shift assay that is designed to optimize conditions for nuclear magnetic resonance studies, which typically require stable samples at high concentration and ambient (or higher) temperature. We demonstrate that for two challenging proteins, the multicomponent screen helped to identify ingredients that increased protein stability, leading to clear improvements in the quality of the spectra. Thermal shift assays provide an economic and time-efficient method to find optimal conditions for NMR structural studies.

**Electronic supplementary material:**

The online version of this article (doi:10.1007/s10858-016-0027-z) contains supplementary material, which is available to authorized users.

## Introduction

Structural biology allows the functional mechanisms of macromolecules to be studied in atomic detail, providing insight into their function. To achieve these goals, researchers use a range of complementary structural and molecular biology techniques, including DNA manipulation, protein expression and purification, and structure determination by X-ray diffraction, electron microscopy and nuclear magnetic resonance (NMR) spectroscopy. X-ray crystallography and NMR have been extensively used to determine high-resolution structures of proteins and have yielded 100,000 and 10,000 structural database entries, respectively (http://www.rcsb.org/pdb/statistics/). Both these techniques require pure, homogenous and highly concentrated protein samples, conditions that may lead to non-native folding or aggregation. NMR spectroscopy presents additional challenges, as proteins need to be ^15^N, ^13^C and/or ^2^H labeled. This requirement increases the stress on the recombinant organism producing the protein, which, in turn, decreases the yield and leads to high production costs. In addition, sample stability, monodispersity and solubility have a profound effect on the quality of the spectra, which highlights the need to assess protein quality prior to structural characterization.

The common workflow of protein preparation and characterization for NMR includes biophysical techniques such as size exclusion chromatography, dynamic light scattering, mass spectrometry, circular dichroism spectroscopy and analytical ultracentrifugation. Use of these techniques ensures that the protein is correctly folded, pure and monodisperse (see review Raynal et al. 2014). The production of high quality samples is not a trivial task; for example, the stability and homogeneity of proteins are strongly influenced by the chemical environment during protein purification and storage. Moreover, NMR spectroscopy involves long experimental times at temperatures between 20 and 40 °C, which poses an additional demand on the thermal stability of the sample. In this scenario, it is critical to test several buffers in order to identify stabilizing conditions prior to the NMR measurements. This can be achieved with high-throughput using a thermal shift assay, also known as ThermoFluor^®^.

Initially developed for drug discovery (Pantoliano et al. 2001), the application of thermal shift assay screens has been considerably extended in the last decade and the method has become one of the gold standards for rapid assessment of protein stability. The thermal shift assay monitors protein unfolding upon thermal denaturation using a fluorescent, hydrophobic dye as a reporter (Pantoliano et al. 2001). The dye has high affinity for the hydrophobic protein regions, which are typically exposed as the protein unfolds; the method and detailed protocols have been described previously (Niesen et al. 2007; Pantoliano et al. 2001; Vivoli et al. 2014). A thermal shift assay can test systematic variations of buffers and/or solubility enhancers (salts, amino acids, sugars, polyols and reducing reagents) and has been applied successfully in processes such as optimization of purification protocols (Boivin et al. 2013), detection of ligand interactions (Kranz and Schalk-Hihi 2011; Niesen et al. 2007), to decrypt proteins of unknown function and modes of action (Carver et al. 2005; Lea and Simeonov 2012), and for optimization of conditions for crystallization (Dupeux et al. 2011; Ericsson et al. 2006) or electron microscopy (Chari et al. 2015).

In this work we attempt to use protein thermal stability as a measure of sample quality for NMR studies. The factors determining the quality of protein preparations for NMR are different from crystallography. NMR studies can tolerate a limited level of heterogeneity; however, solubility, monodispersity and stability at high concentrations and temperatures >15–20 °C remain crucial requirements. In addition, the NMR signals of the buffers should not interfere with the signals of the protein. To satisfy these requirements, we designed a specialized ThermoFluor screen that has been tailored to improve the sample conditions for NMR studies. We demonstrate that the screen affords significant improvements in either solubility or fold stability for two challenging proteins, where initial experiments in standard buffer conditions showed spectra of poor quality.

## Materials and methods

### Protein production

The codon optimized sequence for expression in *E. coli* for *Saccharomyces cerevisiae* RTT109 was obtained from Geneart. Full length RTT109 was sub-cloned for expression into pETM-11, containing an N-terminal His6-tag followed by a Tobacco Etch Virus (TEV) protease cleavage site. Per-deuterated and selectively ^1^H, ^13^C methyl labeled RTT109 (Met-[^13^CH_3_]^ε^, Val-[^13^CH_3_]^γproS^, Leu-[^13^CH_3_]^δproS^, Ile-[^13^CH_3_]^δ^) was obtained by expression in *E. coli* BL21 (DE3) grown in M9 minimal medium in 100 % D_2_O. For selective labeling, precursors from the QLAM-I^δ1^/M^ε^/LV^proS^ kit (nmr-bio) were used following the manufacturer’s instructions. Protein production was induced at OD600 = 0.8 by addition of 0.5 mM IPTG and was allowed to continue for 16 h at 16 °C. Cells were harvested by centrifugation, resuspended in lysis buffer (1× PBS, 500 mM NaCl, 10 mM imidazole, 5 mM BME, 1× protease inhibitor (Roche; 11697498001) and lysed by sonication. The protein was purified by Immobilized Metal ion Affinity Chromatography (IMAC, HisTrap HP 5 ml). The IMAC column was equilibrated and washed with buffer A (25 mM TRIS pH 7.5, 600 mM NaCl, 5 mM 2-mercaptoethanol, 20 mM imidazol) and the protein was eluted using a 20 column volume (CV) gradient to 100 % buffer B (25 mM TRIS pH 7.5, 600 mM NaCl, 5 mM 2-mercaptoethanol, 500 mM imidazol). RTT109 was further purified by gel filtration (Superdex S200 16/60) in a buffer containing 25 mM TRIS pH 7.5, 600 mM NaCl, 5 mM 2-mercaptoethanol.

Protein BA was cloned into the pETM-44 vector with a hexa-histidine maltose-binding protein (MBP) tag. The BA plasmid was a generous gift from the Helmholtz-Institute for Pharmaceutical Research Saarland (HIPS), Saarbruecken. Uniformly ^15^N labeled BA was obtained by growth in M9 minimal media containing ^15^N-ammonium chloride as the sole nitrogen source. Cells were induced with 0.1 mM IPTG at an OD600 = 0.6 and subsequently grown at 16 °C for 18 h. Clarified cell lysates in buffer containing 50 mM TRIS, 0.5 M NaCl, pH 8.0 (Buffer A) were applied to HisTrap 5 ml columns (GE Lifesciences) equilibrated in the same buffer and eluted with a linear gradient from 0 to 500 mM imidazole in 4 CVs. The fusion tag was then cleaved by overnight treatment with His-HRV3C protease at 4 °C in Buffer A. A final purification step was carried out by size exclusion chromatography (Superdex S75 16/60) in a buffer containing 50 mM TRIS, 150 mM NaCl, pH 8.0.

### Preparation of a ThermoFluor buffer screen optimized for NMR studies

**Fig. 1:**
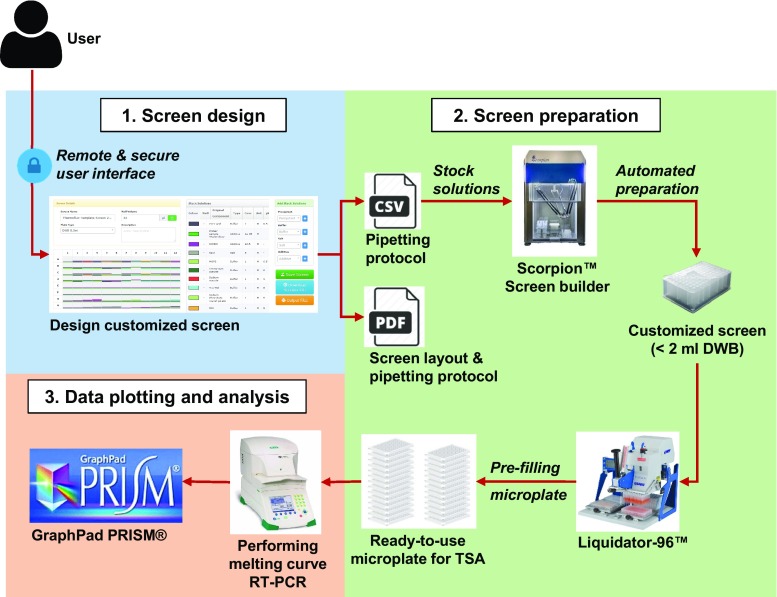
Description of the ThermoFluor screen platform workflow of the SPC facility at EMBL-Hamburg. The SPC facility hosts a platform to design and prepare an optimized screen for ThermoFluor, which includes (1) a web interface to design the screen composition remotely, (2) a liquid handling robot for the automated formulation of the screen, and (3) a data analysis interface

A web interface was developed within the Crystal Information Management System (CRIMS, www.crims-project.org) for the design of custom-made buffer screens in a 96-well format. The interface generates a worksheet with (1) a screen layout comprising a detailed well composition, (2) a table with all components required, comprising a calculation spreadsheet, and (3) a pipetting protocol for each well. The pipetting protocol can be formatted into scripts for a liquid handling robot, such as a Scorpion™ (Art Robbins Instruments) at the Sample Preparation and Characterization (SPC) facility located at the EMBL@PETRA3 synchrotron (Boivin et al. 2016) (http://www.embl-hamburg.de/services/spc). A library of stock solutions was prepared and each solution was filtered before use. To set the pH of the buffers, appropriate amounts of an acid and its conjugate base were prepared according to the Henderson-Hasselbalch equation. The customized screen was formulated at 1.2× concentrated solution in a 2 ml deep well block to allow further dilution with the protein and the dye for the thermal shift assay. The workflow of the platform is summarized in Fig. 1. Researchers who would like to take advantage of this platform should communicate directly with the SPC staff (spc@embl-hamburg.de).

### Performing thermal shift assays

The general approach and protocols have been described previously (Boivin et al. 2013). In short, the PCR microplate (MicroAmp Fast Optical 96-Well; 4346906; Live Technologies) was prefilled with 21 μl of solution per well from the pre-made thermal shift assay screens (1.2× concentrated) using the manual pipetting Liquidator96™ (Mettler Toledo). Then, we added to each well 2 μl of purified RTT109 or BA at 1.6 mg/ml (30 μM) and 1.25 mg/ml (25 μM) respectively, followed by 2 μl of diluted SYPRO Orange solution (ThermoFischer Scientific, S-6651) at 62.5× in water to reach the final volume of 25 μl. Thus, the initial sample buffer was diluted 12.5 times in the assay buffer. The microplates were sealed with an adhesive optical clear seal (MicroAmp Optical Adhesive Film) and centrifuged before being heated using an RT-PCR instrument (StepOnePlus Real-Time PCR System, Applied Biosystems). Data were collected using the channel for the JOE^(^™^)^ dye. The fluorescence in each well was measured at regular intervals with a temperature gradient of 1.2 °C per minute over a temperature range spanning typically from 4 to 80 °C. The interaction of the dye with the molten protein leads to a sigmoidal curve. The temperature at which 50 % of the protein is unfolded and bound to the fluorescent dye (apparent melting temperature, T_m_) corresponds to the inflexion point of the slope. Raw data were plotted and analyzed using GraphPad PRISM (http://www.graphpad.com). The transition midpoint was calculated automatically using an in-house written script and the calculations were verified individually (Fig. 1). The T_m_ value is reported for each condition (Figs. S1, S2).

### NMR spectroscopy

NMR experiments were acquired on Bruker Avance 800and 850 MHz spectrometers equipped with HCN triple-resonance cryoprobes. All experiments were performed at 298 K. RTT109 was present at a concentration of 25 μM in both buffer conditions (optimized and non-optimized, see “Results and Discussion” section) in 99 % D_2_O. ^1^H–^13^C HMQC experiments were recorded for the protein RTT109 with 128 transients each, 3072 (^1^H) × 128 (^13^C) complex points, and acquisition times of 86 and 15 ms in the ^1^H and ^13^C dimensions, respectively, giving a total experiment time of ~6 h. Protein BA was present at a concentration of 50 μM in the standard NMR buffer and at 150 μM in the optimized buffer. Two-dimensional ^1^H–^15^N TROSY-enhanced correlation spectra were acquired on uniformly ^15^N-labeled samples of BA in both buffer conditions with a data size of 1024 (^1^H) × 100 (^15^N) complex points and 36 ppm spectral width in the indirect dimension. The experiments were signal-averaged over 128 transients for ~12 h. The BA data were processed and analyzed using NMRPipe/NMRDraw (Delaglio et al. 1995). The RTT109 data were processed in Topspin (Bruker) and analyzed using CCPNMR analysis (Vranken et al. 2005).

## Results and discussion

### Design of a buffer screen for NMR

After protein expression and purification, the buffer conditions of the final NMR sample should be optimized to avoid protein aggregation, slow precipitation or degradation (Bagby et al. 2001). Thermal shift assays have been developed to support protein purification, crystallization and electron microscopy (see “Introduction” section). However, many of the conditions included in these screens are not suitable for NMR studies, which require buffers with low salt, a limited pH range and no additives that interfere with the NMR measurements. Here, we describe a buffer screen that has been optimized to identify stabilizing conditions compatible with NMR studies. We screen 96 conditions with variable buffer compositions, salt concentrations and additives. The goal is to identify conditions for which the protein fold is most stable as well as to prevent protein aggregation and increase overall protein solubility. We selected 14 different buffers that preserve high sensitivity of NMR measurements (Kelly et al. 2002). Two types of buffer components are included in the screen: (1) buffers with no detectable protons, such as sodium or potassium phosphate (pH 5.0–7.5), which perform best in homonuclear NMR experiments; (2) protonated buffers, which are either available in deuterated form or compatible with heteronuclear NMR experiments. The buffers have been chosen based on their p*K*a value. Protein stability is commonly optimal at physiological pH 6.0–7.5; if possible, protein NMR experiments are carried out at slightly acidic pH to reduce the chemical exchange rate of the amide protons with water; at basic pH, the intensity of the H_N_ signals drops, due to fast exchange with the solvent. In our screen we include buffers with pH values between 5 and 8, as a compromise between protein stability and spectra quality. The buffer concentration is kept constant at 50 mM, except for sodium phosphate pH 7.0. This buffer is preferred in NMR, and a concentration range of 20–200 mM is screened for the most stabilizing condition.

**Fig. 2:**
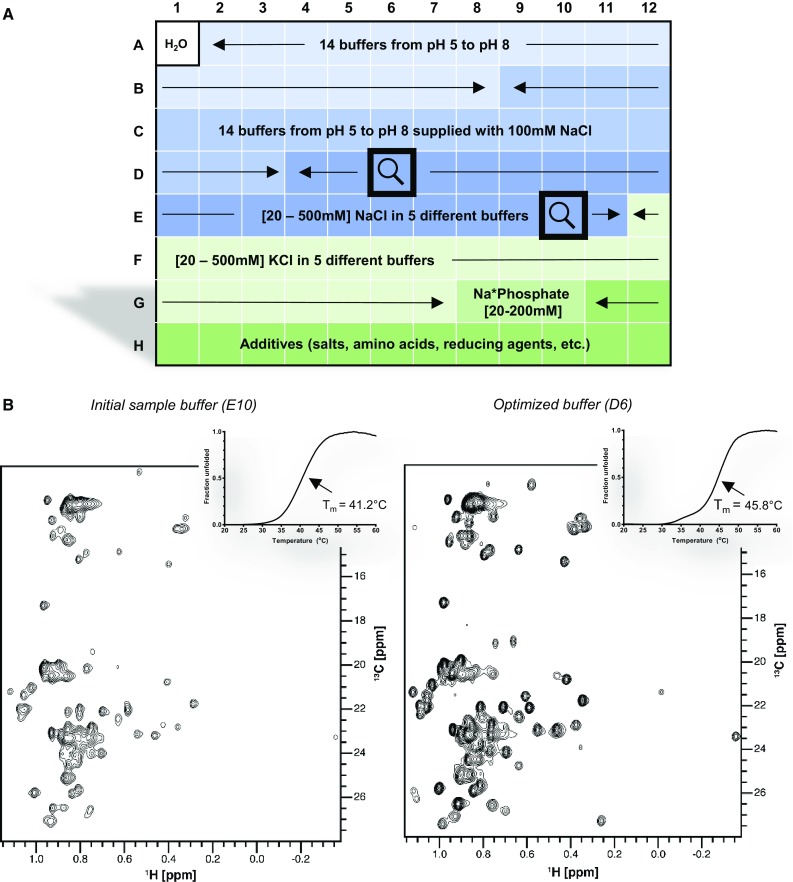
Optimization of sample conditions using the ThermoFluor screen improved NMR spectrum quality. **a** A ThermoFluor screen designed to perform protein profiling by thermal shift assay using a matrix-based approach. **b** Comparison of the ^1^H–^13^C-methyl TROESY spectra of RTT109 recorded before (*left*) and after (*right*) optimization. The *inset* shows representative melting curves in the corresponding buffer (E10; 50 mM sodium phosphate pH 7.0, 200 mM NaCl, D6; 50 mM sodium citrate pH 5.5, 200 mM NaCl). A complete description of the screen composition is available in supplementary Fig. S1 and the corresponding T_m_ values are displayed in Fig. S2. 1 mM DTT was added to the NMR samples in both buffers to prevent oxidation of exposed cysteines

For NMR studies, the biomolecule needs to be stable at concentrations >50 μM and at temperatures ≥20 °C. For proteins displaying a charged surface, solubility increases with ionic strength, while the intrinsic signal-to-noise of the experiment is worse in high salt. To determine the minimum ionic strength necessary to prevent aggregation and/or increase solubility, without compromising the sensitivity of the NMR experiments, we screen NaCl and KCl gradients from 20 to 500 mM in five buffers with pH 5.5–7. In addition, we integrate in the screen reducing reagents such as 5 mM DTT and 2 mM TCEP, which are commonly used to prevent protein aggregation via oxidation of surface cysteines. Other reagents may also contribute to protein stability and solubility, such as non-denaturing detergents (Octyl-glocoside, CHAPS) and amino acids (Anglister et al. 1993; Boivin et al. 2013). A schematic representation of the screen layout is shown ( Fig. 2a) and a detailed description can be found in the supplementary section (Fig. S1).

### Improving protein thermal stability and NMR sensitivity

The customized “NMR” ThermoFluor screen was used to optimize solution conditions for proteins that in initial trials were either poorly soluble or insufficiently stable for NMR experiments. One such case is the *Saccharomyces cerevisiae* histone acetyltransferase (HAT) Regulator of Ty1 Transposition gene product 109 (RTT109) (Han et al. 2007a). RTT109’s acetylation activity and specificity are regulated by association with different histone chaperones, such as Anti-silencing function 1 (Asf1) and Vacuolar protein sorting 75 (Vps75) (Han et al. 2007b; Park et al. 2008). Crystal structures of RTT109 (Lin and Yuan 2008; Stavropoulos et al. 2008) and different RTT109–VPS75 complexes (Su et al. 2011; Tang et al. 2011) are available; yet, the mechanism of activation and structural understanding of the RTT109-substrate interaction remains elusive (D’Arcy and Luger 2011). Here, we set out to study the interaction of RTT109 with its protein partners, including histones, in multi-molecular complexes.

RTT109 can be expressed and purified in phosphate or TRIS buffer with high salt concentrations (>500 mM NaCl); however, extensive precipitation and low signal-to-noise were observed in initial NMR experiments. To improve the sample stability, we used a custom designed thermal shift assay, comparing 96 different buffer conditions in parallel. The large number of assayed conditions enabled the identification of general trends in the effect of different components on protein stability. In agreement with the need for high concentrations of sodium chloride in the purification buffers, the thermal shift assay revealed higher protein stability at high salt concentration in all tested solutions. In 50 mM phosphate buffer at pH 7.0, for example, an increase of the melting temperature of almost 10 °C from 37.9 to 47.3 °C was observed upon increasing the salt concentration from 20 to 500 mM NaCl. A notable exception was 50 mM sodium citrate pH 5.5, where the T_m_ was consistently high and nearly independent of salt concentration. Indeed, when comparing 50 mM sodium citrate pH 5.5 containing either 20 or 500 mM NaCl, only a 2 °C difference in T_m_ is observed. Intrigued by the stabilizing effect of citrate, we recorded ^1^H–^13^C HMQC spectra of methyl labeled RTT109 in 50 mM sodium citrate pH 5.5 and 50 mM sodium phosphate pH 7.0, both with 200 mM NaCl (Fig. 2b). A low protein concentration (25 μM) was used to circumvent protein precipitation in the phosphate buffer. Marked improvements in signal-to-noise and a more homogenous distribution of peak intensities across the spectrum were observed in the citrate buffer. In addition, the number of well-resolved detectable peaks increased modestly from 59 to 63, out of 73 expected peaks in the Leu, Val region, and from 23 to 26, out of 26 expected peaks in the Ile region of the spectra. The observed effects can be attributed to an improvement in conformational stability of RTT109 in sodium citrate. It is well-known that buffer components can affect chemical exchange in proteins (Wong et al. 2013); in this study the presence of citrate seems to stabilize RTT109 in a distinct conformation.

### Improving protein solubility and suitability for NMR

In another case, screening of NMR solution conditions by thermal shift assay helped to improve protein solubility. Protein “BA” (molecular weight, 56 kDa) is an adenylation domain of a non-ribosomal peptide synthase (NRPS). Non-ribosomal peptide synthases are multi-domain, mega-polypeptides that produce a variety of pharmacologically active peptides (Finking and Marahiel 2004). Adenylation domains within the NRPSs are responsible for substrate selection, i.e. recognition of the building blocks that make up the peptides. Detailed understanding of the structure and interactions of this adenylation domain with other catalytic domains of the NRPSs should benefit the engineering of novel peptides.

Even though the protein BA could be expressed and purified with high yields, NMR structural investigation was hampered by low protein solubility and poor spectral quality. Initial studies were conducted in standard NMR conditions, with 50 mM sodium phosphate as the buffering agent at pH 7.0 and 50 mM NaCl. The maximum concentration that could be achieved in these conditions was <3 mg/ml, as the protein was prone to aggregation followed by substantial precipitation. This presented a major bottleneck in studying BA by NMR.

**Fig. 3:**
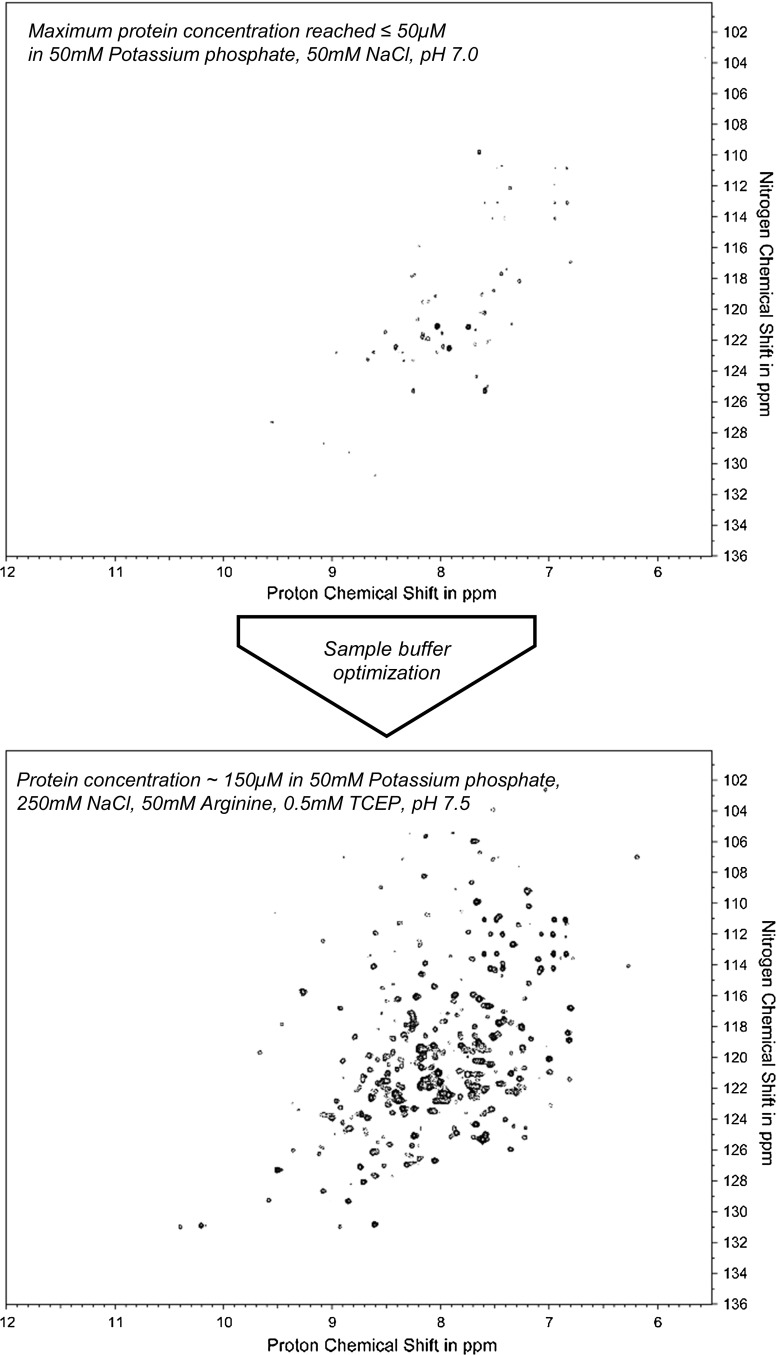
Optimizing protein solubility for NMR studies. A comparison of the ^1^H–^15^N correlations of the BA protein in the initial buffer conditions (*upper panel* 50 mM potassium phosphate pH 7.0, 50 mM NaCl) and in the optimized buffer conditions (*lower panel* 50 mM potassium phosphate pH 7.5, 250 mM NaCl, 50 mM arginine, 0.5 mM TCEP). The maximum protein concentration that could be reached in the initial buffer did not exceed 3 mg/ml, while in the optimized conditions the protein could be concentrated to 9 mg/ml

Protein profiling was carried out using a 96-well plate thermal shift assay to identify buffer conditions that are potentially beneficial for NMR studies. Higher melting temperatures were observed for the phosphate buffer at pH 7.5. Moreover, the addition of arginine increased the thermo-stability of BA. The addition of arginine and glutamate mixtures to diluted protein samples has been previously shown to reduce aggregation during sample concentration (Golovanov et al. 2004). With these observations in mind, an optimized sample buffer was designed with the goal of limiting aggregation and increasing both protein solubility and stability. The new sample buffer (50 mM potassium phosphate, 50 mM arginine, 250 mM NaCl, 0.5 mM TCEP, pH 7.5) yielded a threefold improvement in solubility, enabling us to achieve a protein concentration suitable for NMR measurements (9 mg/ml, 150 μM). Figure 3 compares the ^1^H–^15^N correlation spectra in the initial and optimized conditions. The latter spectrum is clearly superior, showing good signal-to-noise ratio and excellent chemical shift dispersion. This example demonstrates that minor changes in solution conditions can have a sizeable effect on protein solubility. Thus, a high-throughput thermal shift screen designed for NMR, requiring very low amounts of protein, provides an efficient way to rapidly optimize the sample conditions for solution NMR measurements.

**Fig. 4:**
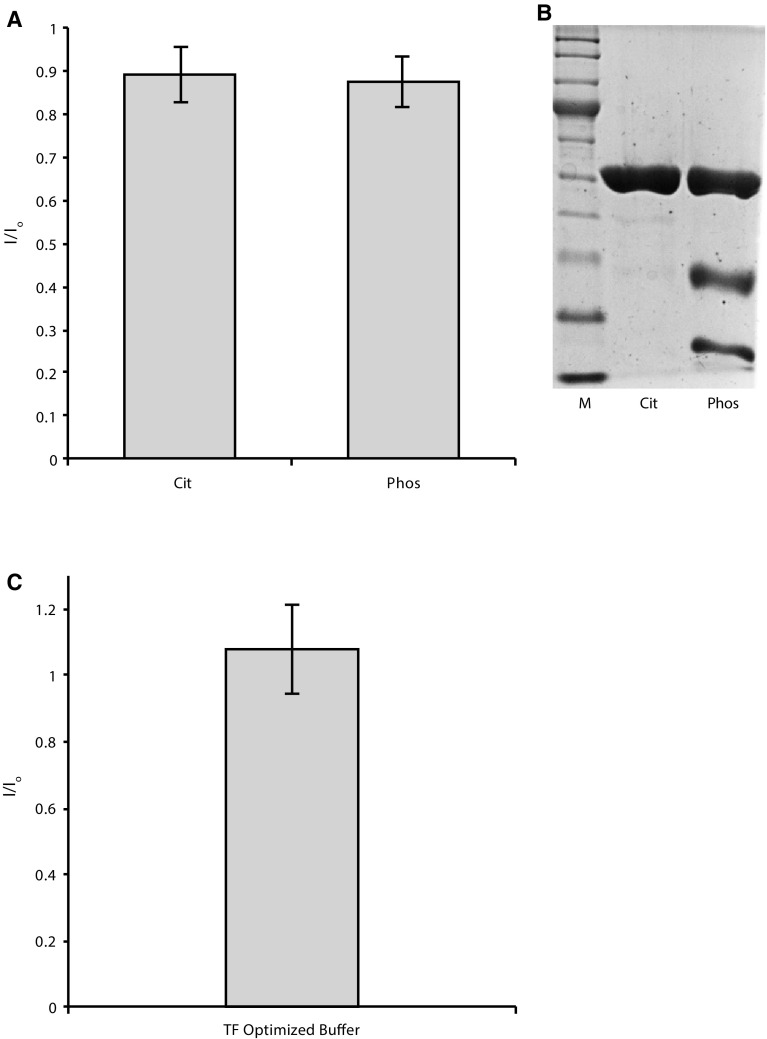
Protein long-term stability. **a** Average of the intensity ratios at Day 1 and Day 10 for each peak in the ^1^H–^13^C-methyl TROESY spectrum of Rtt109 in the initial buffer (Phos) and in the optimized buffer (Cit). Both samples show only a modest intensity loss. **b** Gel page of Rtt109 after 10 Days in the initial (Phos) and optimized (Cit) buffer. Digestion of Rtt109 in the initial buffer is evident. **c** Average of the intensity ratios at Day 1 and Day 10 for each peak in the ^1^H–^15^N-TROESY spectrum of BA in the optimized buffer. No intensity loss is observed

For the two cases tested here, thermal stability correlated also with long-term stability, as both Rtt109 and BA were stable in the optimized buffers for over 10 days (Fig. 4). Contrarily, Rtt109 was degraded over time in phosphate buffer (Fig. 4b). As a note of caution, this correlation is purely empirical and may not hold for all proteins.

## Conclusions

Thermal shift assays provide a fast and high-throughput method to screen protein stabilizing conditions for specific applications. Here, we have developed a thermal shift assay screen that was optimized to meet the requirements of NMR-based structural studies. The NMR-specific thermal shift assay allowed a notable improvement in spectral quality for two unstable and poorly soluble proteins. The development of this screen has been facilitated by the open-access SPC facility’s liquid handling platform at EMBL-Hamburg. We believe that this approach can be broadly used to optimize sample preparation for distinct purposes where sample quality remains the major bottleneck.

## Electronic supplementary material

Below is the link to the electronic supplementary material.

Fig. S1 **NMR Schematic screen layout**. The screen was prepared as (1.2X) stock plates. The final concentration for the assay is displayed in the table. Each solution was individually adjusted to the designated pH. Typical assay protocols involved dispensing 21 μl of relevant (1.2X) stock solutions into assay plates, followed by the addition of 2 μl of protein solution, and 2 μl of SYPRO Orange working concentration (62X). RT-PCR was programmed to ramp the temperature from 4°C to 80°C at 1.2°C/min (PPTX 46 kb)

Fig. S2 **Results of ThermoFluor assay of RTT109 (A) and BA (B) using the NMR optimized screen**. The position of the wells corresponded to Fig. S1. Heat map colors code for the melting temperature in degrees Celsius (the temperature increases from light blue to dark red). The values of the apparent melting temperature are reported for each condition. nd stands for “not determined”, indicating that the melting curve could not be evaluated with confidence. The precision in T_m_ determination is generally ± 0.2°C (PPTX 128 kb)

## Abbreviations

HTP: High-throughput
T_m_: Melting temperature
NMR: Nuclear magnetic resonance
RT-PCR: Real-time quantitative reverse transcription
SPC: Sample preparation and characterization
