# Supplementary figures and images for: Optimization of protein samples for NMR using thermal shift assays
Source: J Biomol NMR. 2016 Mar 17;64:281–9. doi: 10.1007/s10858-016-0027-z (PMC4869703; doi:10.1007/s10858-016-0027-z)

## Slide 1
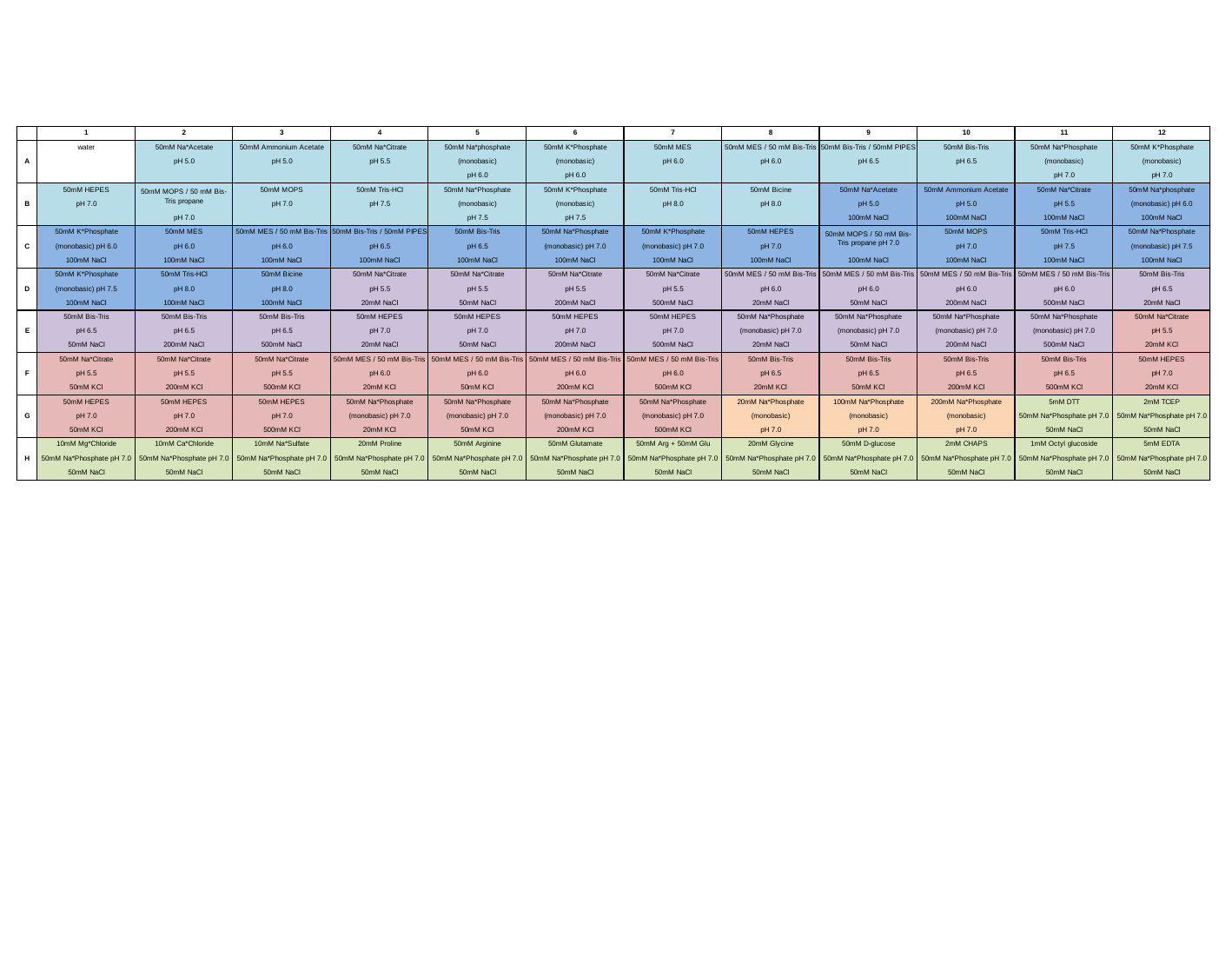

Supplement: Supplementary file 1 — NMR Schematic screen layout. The screen was prepared as (1.2X) stock plates. The final concentration for the assay is displayed in the table. Each solution was individually adjusted to the designated pH. Typical assay protocols involved dispensing 21 μl of relevant (1.2X) stock solutions into assay plates, followed by the addition of 2 μl of protein solution, and 2 μl of SYPRO Orange working concentration (62X). RT-PCR was programmed to ramp the temperature from 4°C to 80°C at 1.2°C/min (PPTX 46 kb) [file 10858_2016_27_MOESM1_ESM.pptx]

## Slide 1
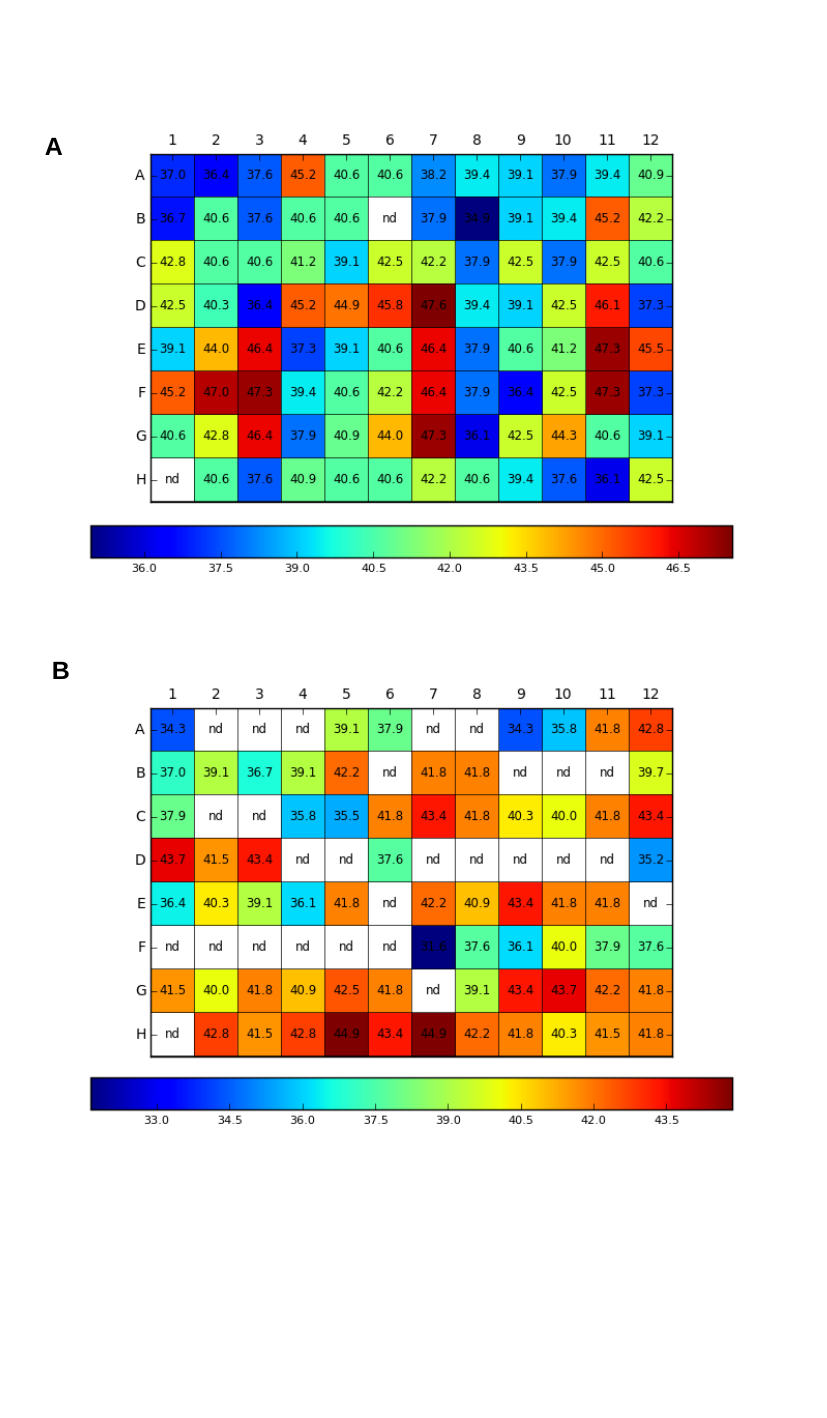

A
B

Supplement: Supplementary file 2 — Results of ThermoFluor assay of RTT109 (A) and BA (B) using the NMR optimized screen. The position of the wells corresponded to Fig. S1. Heat map colors code for the melting temperature in degrees Celsius (the temperature increases from light blue to dark red). The values of the apparent melting temperature are reported for each condition. nd stands for “not determined”, indicating that the melting curve could not be evaluated with confidence. The precision in Tm determination is generally ± 0.2°C (PPTX 128 kb) [file 10858_2016_27_MOESM2_ESM.pptx]
